# The biopsychosocial processes in autism spectrum disorder

**DOI:** 10.1186/1755-7682-6-22

**Published:** 2013-05-08

**Authors:** Edgar Bittner Silva, Rosangela Filipini, Carlos Bandeira de Mello Monteiro, Vitor E Valenti, Sionara Melo Figueiredo de Carvalho, Rubens Wajnsztejn, Maria do Carmo Andrade Duarte de Farias, Cícero Cruz Macedo, Luiz Carlos de Abreu

**Affiliations:** 1 Laboratório de Delineamento de Estudos e Escrita Científica, Faculdade de Medicina do ABC, Av. Príncipe de Gales, 821, 09060-650, Santo André, São Paulo, Brazil; 2 Escola de Artes, Ciências e Humanidades da Universidade de São Paulo, Av. Arlindo Béttio, 1000 Ermelino, Matarazzo, São Paulo SP CEP: 03828-000, Brazil; 3 Departamento de Fonoaudiologia, Faculdade de Filosofia e Ciências, Universidade Estadual Paulista, UNESP, Av. Hygino Muzzi Filho, 737, Marília SP CEP: 17525-900, Brazil; 4 Universidade Federal de Campina Grande, R. Aprígio Veloso, 882, Cajazeiras PB CEP 58900-000, Brazil; 5 Departamento de Pediatria. Faculdade de Medicina de Barbalha, CE. Universidade Federal do Ceará, Av. da Universidade, 2853, Fortaleza CE, CEP: 60020-181, Brazil

**Keywords:** Autistic disorder, Mental disorder, Central coherence, fMRI

## Abstract

**Background:**

Autism is a disorder characterized by pervasive social and communicative impairments, repetitive and stereotyped behaviors and restricted interests. Its causes and effects have been researched from various neurocognitive theoretical perspectives and with the aid of neuroimaging technology. We aimed to describe biopsychosocial processes characteristic of the Autism Spectrum Disorders.

**Method:**

Literature review using Medline and Scopus databases published between 2001 and 2011, with the keywords "autism", "theory of mind", "executive functions", "central coherence" and “fMRI”.

**Results:**

The studies found were plotted and organized into tables and an explanatory diagram of the main findings was produced.

**Conclusions:**

The most popular neurocognitive theories are still unable to fully explain the characteristics of the complications that autistic spectrum disorder causes to the quality of life of individuals living with autism. The association of clinical research and neuroimaging may contribute to a better understanding of the functioning of the brain affected by the disorder.

## Background

The Autistic Spectrum Disorders encompass conditions known as Autistic Disorder, Asperger Syndrome and Pervasive Development Disorder - Not Otherwise Specified (PDD-NOS), which is diagnosed based on behavior with differentiation determined by the presence or absence and intensity (dimension) of symptoms. The diagnostic criteria stipulate the age of about 3 years as the basis its identification, but some signals are already identifiable before that [1].

The etiology of autism is still largely unknown. Despite the consensus about the biological and genetic basis of the disorder, it is believed that many genes and causes contribute to the construction of the condition [1,2].

The neurocognitive theories such as the Theory of Mind which is the ability to analyze the world from the perspective of the other, the Executive Functions the level of control the individual exercises over his/her behavior, memory and attention and Central Coherence, the ability to integrate perceived information into a coherent whole [2] have contributed to a better understanding of the symptoms of the disorder, especially the social deficit and its roots.

In this review we aimed to describe advances in the study of biopsychosocial process of people diagnosed with autism.

## Method

The Medline database (via PubMed) and Science Direct (via Scopus) were searched using the following keywords: "autism", "theory of mind", "executive functions", "central coherence" and "fMRI". We used the "related articles" in PubMed (U.S. National Library of Medicine National Institutes of Health), which allowed us to get the references of studies recovered during our research.

Publications were included in the analysis if any of their titles or abstracts were available in English or Portuguese. The review had its onset in July 2011 and was completed in December 2011. Publications were excluded if published before 2000. Other studies of autism that offered additional relevant information found in the same database were also examined. Each publication was reviewed to identify the author, study period, objective and main contributions to the theme.

**Figure 1:**
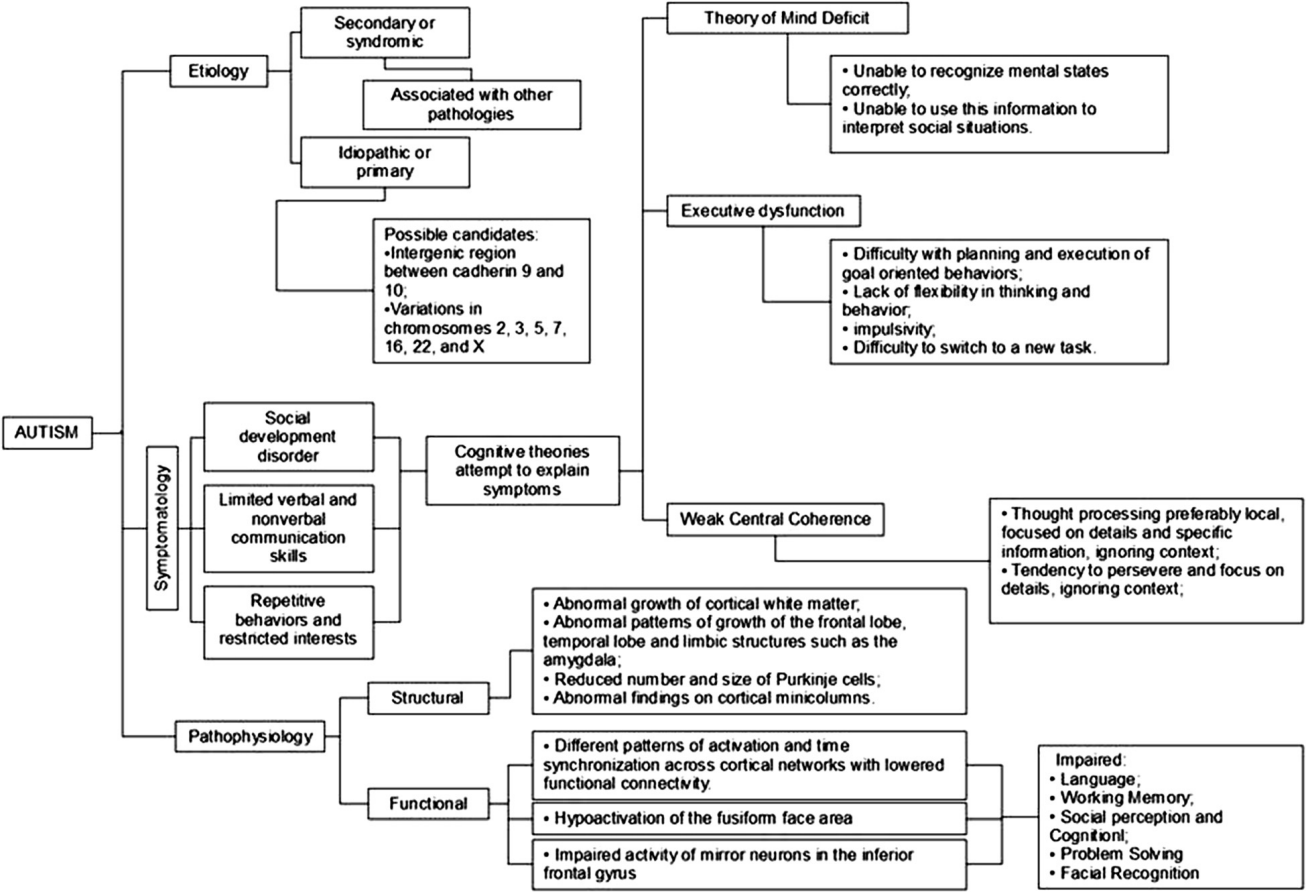
Conceptual framework of Autism.

The data were plotted and organized into tables with the types of selected studies and their indexing (Table 1) and the summaries of the papers found on neurocognitive explanatory theories of autism (Table 2) and findings concerning the neurobiology of autism (Table 3) leading to the production of a conceptual framework (Figure 1).

**Table 1:** Publications and types of study related to the biopsychosocial process in Autism

**Type of study**	**Medline**	**JCR-ISI**	**SJR-Scopus**
Review	5	6	5
Comparative Study	11	10	10
Experimental	10	10	10
Clinical Trial	2	1	2

Medline: Medical Literature Analysis and Retrieval System Online.

JCR-ISI: Journal Citation Reports.

SJR-Scopus: SCImago Journal Rank.

**Table 2:** Summary of articles related to neurocognitive explanatory theories of Autism

**Author, date**	**Conclusions**	**Contributions**
Pisula, 2010 [2]	Despite the progress, none of the theories can fully explain the neurocognitive complexity and impact of symptoms characteristic of ASD on the development of the individual.	Sheds light on the importance of research on the interaction between the various explanatory theories of autism among themselves and with individual characteristics.
Beaumont, Newcombe, 2006 [3]	Difficulties in the attribution of mental states by adults with AS cannot be solely attributed to weak central coherence, highlighting the need of taking into account aspects related to the ToM deficit.	Identifies differences between AUT and AS regarding the ToM and CC.
Miller, 2006 [4]	Taking ToM into consideration the may help clinicians improve communication and language development of children.	Demonstrates the importance of ToM for language development, contributing for the practice on speech therapy.
Silani et al., 2008 [5]	Difficulties in emotional awareness are related to a hypoactivation of the anterior insula in individuals with AS and in people with TD, and particular difficulties in emotional awareness in individuals with AS are not related to impairments in self-reflection/mentalizing.	Finds evidence that alexithymic symptoms are usually mediated emotional responses of second order.
Yang et al., 2009 [6]	ToM is significantly correlated with inhibitory control. The performance on tasks of inhibitory control did not affect performance on ToM tasks.	Explores the relationship between EF and ToM emphasizing the role of inhibitory control.
Bogte et al., 2008 [7]	The cognitive flexibility in people with high-functioning autism is similar to people with TD in simpler tasks, but with longer response time.	Investigates the relevance of slowness of cognitive processes in the functionality of the person with AUT.
Geurts et al., 2004 [8]	Children with HFA exhibit more widespread and deep problems in EF tasks than children with ADHD.	Reveals traits and executive dysfunctions shared among people with ADHD and HFA.
van Lang et al., 2006 [9]	Adolescents with intellectual disability and comorbid ASD have CC weaker than people with equivalent age and IQ.	Presents CC as a possible tool for differential diagnosis between Intellectual Disability with and without comorbid AUT.
Noens, van Berckelaer-Onnes, 2004 [10]	A wealer CC implies problems in making sense of the world and hence of communication in people with ASD and intellectual disability.	A better understanding of CC can assist in developing communicative focused individual interventions.
Happé et al., 2006 [11]	Findings suggest deficits in EF are less severe and persistent in people with AUT than people with ADHD.	Describes the existence of different profiles for the deficits in EF for people with ADHD and AUT and shows improvement of the deficits with age and intervention.
Luna et al., 2007 [12]	While executive dysfunction is present throughout development, there is evidence for developmental progressions of EF in AUT.	Highlights the need for age-specific interventions aiming at improving the cognitive abilities of individuals with AUT.
Robinson et al., 2009 [13]	People with ASD exhibit a specific pattern of executive dysfunction, difficulties with planning, inhibition of prepotent responses and self-monitoring that can vary with age.	Proposes a multidimensional notion of EF, with difficulties in planning, inhibition of prepotent responses and self-monitoring traits reflecting the ASD that are independent of IQ and verbal ability, and relatively stable throughout childhood.
López et al., 2008 [14]	Did not find a significant positive relationship between global and semantic processing in children with autism and children with TD.	Findings show that the CC is not a unitary construct, and may be composed of various skills, and indicate the possibility of subtypes of AUT.
Teunisse et al., 2001 [15]	Although not universal in the AUT, a weak CC and poor cognitive flexibility are significantly more common in people with AS than in those with TD.	The weak CC does not seem to be related to the severity of symptoms of AUT.
Belmonte, 2009 [16]	ToM dysfunction is not universal in AUT, and is preceded in the development and predicted by abnormalities of attention, EF and language.	Recognizes the importance of ToM, but emphasizes the relevance of other cognitive functions and social development.
Jarrold et al., 2000 [17]	There is a relationship between individual differences in CC and the development of a ToM.	Relates the development and interactions, in individuals with AUT, of apparently independent cognitive mechanisms.
Rajendran, Mitchell [18]	Advances in different paths have led researchers to understand AUT as a complex condition dependent on individual, qualitative differences.	Highlights how the understanding of AUT has changed over time and takes into consideration the possibility and implications of recognizing AUT as a neurodevelopmental condition rather than a disorder.

**Table 3:** Summary of articles concerning brain morphophysiological and functional abnormalities

**Author, date**	**Conclusions**	**Contributions**
Levy et al., 2009 [1]	AUT is not a monogenic disorder, in many individuals may be the result of a complex amalgam of multiple simultaneous genetic variations, and present morphological and functional brain abnormalities.	Highlights the importance of understanding biological markers, patterns of cortical organization and connectivity in advancing the treatment of AUT.
Baron-Cohen et al., 2000 [19]	Unlike those with TD in ToM tests, patients with AS or AUT do not show activation of the amygdala when making mentalistic inferences from the eyes. The amygdala may be one of many abnormal neural regions in AUT.	Highlights the role of the amygdala in the symptoms of ASD.
Castelli et al., 2002 [20]	The physiological cause for the mentalizing dysfunction in AUT can be a bottleneck in the interaction between perceptual processes of higher and lower order.	Relates the difficulties in understanding socially relevant movements in AUT to information processing in the visual cortex.
Brunet et al., 2000 [21]	Attribution of intentions to others is associated with a complex brain activity involving the right medial prefrontal cortex when a nonverbal task is used.	Presents data that validate hypothesis of abnormal brain activation in patients with impaired mentalizing.
Castelli et al., 2000 [22]	The regions responsible for processing information about intentions and the ability to make inferences about the mental states of others may have evolved from the ability to make inferences about the actions of other creatures.	Suggests evolutionary history for the abilities that make ToM.
Pierce et al., 2001 [23]	Compared to typical individuals, autistic "see" faces using different neural systems, unique to each individual.	Experiential factors play an important role in the development of the fusiform facial area, related to the processing faces.
Scholz et al., 2009 [24]	There are neighboring but distinct regions in the right temporo-parietal junction involved in ToM and orientation of attention.	Identifies difficulties in the investigation of brain functions that occupy regions close to each other or overlapping.
Johnson et al., 2007 [25]	There is involvement of frontal and parietal attentional networks and sub-cortical excitatory systems in ADHD and a prefrontal cortex dysfunction in children with HFA.	Provides detailed evidence of dysfunction in sustained attention in ADHD significantly higher than in HFA.
Kana et al., 2007 [26]	The neural circuit linked to inhibition in individuals with ASD is atypically activated and is less synchronized, leaving inhibition to be accomplished by strategic control rather than automatically.	Identifies dysfunction in inhibitory cortical level, being disorganized and desynchronized.
Schultz et al., 2000 [27]	Individuals with ASD demonstrate during facial discrimination, a pattern of brain activity consistent with strategies based on characteristics or traits more typical of the perception of non-facial objects.	Highlights possible dysfunction in processing stimuli related to biological and inanimate objects in ASD.
Dapretto et al., 2006 [28]	A dysfunctional mirror neuron system may underlie the social deficits of autism.	Substantiates hypothesis of the importance of mirror neurons in the development of social functions.
Jones et al., 2008 [29]	The behavior of eye contact is already noticeably compromised in children two years of age with ASD, accompanying the person with autism for life.	Subsidizes and highlights the importance of early diagnosis of ASD.

## Results

In the organization of the data we used the following abbreviations: AUT: Autism, ASD: Autistic Spectrum Disorder; AS: Asperger Syndrome, HFA: High-functioning autism; TD: Typical Development; ToM: Theory of Mind, EF: Executive Functions; CC: Central Coherence; OCD: Obsessive-compulsive Disorder, ADHD: Attention Deficit Disorder and Hyperactivity.

## Discussion

Autism is a spectrum disorder that affects the overall development, characterized by impairments in social development, limited verbal and nonverbal communication skills and repetitive behaviors with restricted interests. When thinking autism or autism spectrum disorders etiology and pathophysiology should be taken into consideration, besides the symptoms [2].

Functionally, there are different patterns of activation and synchronization time across the cortical networks with lowered functional connectivity affecting language, working memory, cognition and social perception and problem solving, hypoactivation of the fusiform facial area [23,26], affecting facial recognition. Deficient activity of mirror neurons in the inferior frontal gyrus has also been reported [28].

From birth, the baby gives preferential attention to the mother or caregiver's face, particularly the eyes, a phylogenetically built-in behavior with an important role in the social development of the child [29]. As early as age two a preference for paying attention to parts of the face which have lower social significance than the eyes can be noticed in individuals with autism.

Unlike what happens with typically developing individuals, who consistently show activation of the fusiform gyrus for this kind of stimulus, people with autism show weak or no significant activation in this area, but show activation of areas not commonly associated with facial recognition [23]. Thus one can say that individuals with autism 'see' faces using neural systems different from those used by people of typical development. Also, it should be noted that each individual uses a unique system for processing the stimulus, reinforcing the notion that the individual experiences play an important role in the neurological development [23].

The characteristic symptoms of autism, particularly the social deficit has received attention from many researchers, with varying success, from three approaches based on neurocognitive theories, namely the Theory of Mind, Executive Dysfunction and Weak Central Coherence [2,18,19].

The Theory of Mind (ToM) relates to our understanding of mental states, whether they are beliefs, desires or knowledge, which allows us to predict or explain the behavior and attitudes of others [17]. In typically developing individuals, nonverbal tasks involving the attribution of intentionality showed activation, leading to increased blood flow, especially in the right inferior and medial prefrontal cortex and also the temporal lobes bilaterally and left cerebellum [20]. The studies reviewed by Castelli et al. [22] also show activities associated with the anterior cingulate cortex, an area in the anterior temporal lobes near the amygdala, and in the superior temporal sulcus (STS) in the temporoparietal junction, having neuroimaging studies shown greater activation in regions including the basal temporal area (temporal gyrus bottom extending to the fusiform gyrus and temporal pole adjacent to amygdala), and the STS in temporo-parietal junction medial prefrontal cortex. In individuals with autism spectrum disorders the authors found decreased activation in this network.

The ability to understand their own emotional states, as well as those of others, yet a consequence of deficits in ToM, is also impaired, which implies consequences on the ability to 'fit in', hampering their participation in social contexts and contributing to the increased rates of depression among these individuals. Even with improvements in self-awareness resulting from experience and motivation, persistent difficulty in learning compensatory strategies in social communication is a barrier difficult to transpose [4,10].

The development of abilities related to ToM, however, also depends on some internal mechanisms linked to the so-called executive functions, cognitive processes that make possible goal oriented complex behaviors that evolve throughout development until mid-adolescence [12]. They include abilities such as planning, working memory, cognitive flexibility, response inhibition and initiation, impulse control and action monitoring [13], seeming to be associated with different regions of the frontal lobes, with connections between the frontal and posterior areas as well as subcortical and thalamic pathways; neural networks along the prefrontal cortex also play an important role in executive functions [12].

Difficulties in planning, inhibition and self-regulation during childhood seem to reflect the symptoms of autism [7,13]. Robinson et al. [13] found poor performance in planning, response inhibition and self-regulation, but preserved mental flexibility in people with ASD compared with TD peers. Bogte et al. [7] found no significant differences between people with high-functioning autism (IQ ≥ 70) and controls regarding cognitive flexibility, finding, however, longer response times for the first when under medication.

Central coherence, another theory that attempts to explain the social deficit in autism is the ability to integrate information into a coherent whole in perceptual and conceptual level [14]. Seeing a wooden object in a rectangular shape, with a pair of doors with locks and handles, one immediately recognizes it as a closet, as well as eyes, nose and mouth in a particular arrangement is a face. When there is a failure in this integrative mechanism it is said that there is a weak central coherence.

A weak central coherence makes it hard for this individual to recognize the stimuli that surround him globally, making the world a series of parts, instead of an integrated whole, as in typical development subjects.

The impaired and limited communication skills of people with autism are characterized by a lack of intentionality and symbol formation, which may indicate that a specific cognitive style underlies the development of deviant communication in autism. The central coherence theory may offer insight into the communication problems specific to people with autism assuming a lesser tendency towards a central coherence leads to problems in trying to make sense of the world and, consequently, communication [10].

In autism information processing seems to be preferably local, rather than global [9], there are indications of excessive preservation of unnecessary connections of short distance and relative lack of long distance connections, leading to low efficiency in information processing that may help explain the tendency to focus on details instead of the whole [7].

The results of Schultz et al. [27] regarding the poor activation of the fusiform gyrus may support the theory of weak central coherence suggesting that, in individuals with autism, processing the stimulus of sight of a face occurs so that attention is paid to details, consistent with the processing of non-facial stimuli, such as objects - elements that depend on specific characteristics for identification, rather than a configurational focus, the set of elements which we call a face [30].

The three aspects studied, however, do not seem to act alone, influencing each other. Jarrold et al. [17] found a relationship between dysfunction in theory of mind and a weak central coherence. The results of Beaumont and Newcombe [3] support the explanation of autism by ToM, also finding evidence of the influence of the central coherence. For Yang et al. [6], ToM was significantly correlated with inhibitory control. Performance on tasks of inhibitory control, however, did not affect performance on tasks ToM.

There are conflicting results. A weak central coherence and poor cognitive switching for Teunisse et al. [15] do not seem to be related to measures of the severity of symptoms, to social understanding and social competence. Even seeming to be significantly more common than in typically developed individuals, a weak central coherence and poor cognitive switching do not seem to be universal for autism. For Belmonte [16], ToM dysfunction is not universal in autism and is developmentally preceded and predicted by changes in attention, executive function and language.

None of these features is exclusive Autism, appearing in other disorders such as ADHD [8,11,25] and also intellectual disability [9], demanding an even more thorough understanding of how these dysfunctions and deficits are manifested specifically in these individuals. Rajendran and Mitchell [18] have also done an extensive review on the subject of both uniqueness and universality of the major cognitive theories coming to similar conclusions.

Advances in studies using positron emission tomography (PET) [20,22] and functional MRI (fMRI) [5,18,24,26,27] have brought new information about the functioning of the autistic brain, providing clues for building programs pharmacological intervention to reduce symptoms and psychosocial programs aiming at increased and improved social participation of the individual with benefits for his/her development.

The findings support Levy et al. [1] which propose, from revised neurological studies seeing of autism as a disorder of cortical organization that causes neuronal deficits in information processing in the nervous system, ranging from organization to synaptic connectivity and brain structure.

An interesting new take on Autism has been provided by the mnesic imbalance theory [31], according to which, an imbalance between declarative and procedural memory may answer for the symptoms of the disorder. Here, a faulty procedural memory, possibly due to brain abnormalities and cerebellar maldevelopment, hinders the development of functions related to ToM, EF and CC and leads to an overuse of declarative memory as compensation. This theory is still disputed as there is a lack of studies on declarative and non-declarative memory in people with moderately low-functioning autism [32].

Understanding Autism as a condition rather than a disorder is also on the spotlight [18]. Findings related to morphology, genetics and the most popular cognitive theories on Autism have led some to see it as a different “way of being”, which may, in the future, have impact on policies, laws and interventions as people with Autism become a vocal group such as the Deaf.

Our study is also interesting for others neurological disorders [30,33-38]. A better understanding of brain functioning and the consequences of autism for individual development may help us develop strategies better attuned to its particularities [10], contributing to the rehabilitation process of the person with autism.

## Conclusion

We conclude that individuals living with autism present symptoms that set them apart from the contemporary social model, reducing their quality of life. None of the neurocognitive theories can fully explain the complications that the disorder brings to the quality of life of individuals with autism. Together they offer essential clues to develop interventions. Neuroimaging studies contribute greatly to a better understanding of the functioning of the brain affected by the disorder.

## Competing interests

The authors declare that they have no competing interests.

## Authors’ contributions

All authors participated in the acquisition of data and revision of the manuscript. All authors conceived of the study, determined the design, interpreted the data and drafted the manuscript. VEV and LCA determined the design and drafted the manuscript. All authors read and gave final approval for the version submitted for publication.
